# Developmental therapeutics for inflammatory breast cancer: Biology and translational directions

**DOI:** 10.18632/oncotarget.13778

**Published:** 2016-12-02

**Authors:** Ricardo Costa, Cesar A Santa-Maria, Giovanna Rossi, Benedito A Carneiro, Young Kwang Chae, William J Gradishar, Francis J Giles, Massimo Cristofanilli

**Affiliations:** ^1^ Developmental Therapeutics Program, Division of Hematology/Oncology, Feinberg School of Medicine, Chicago, United States of America; ^2^ Robert H. Lurie Comprehensive Cancer Center of Northwestern University, Chicago, United States of America

**Keywords:** inflammatory breast cancer, targeted therapy, immunotherapy

## Abstract

Inflammatory breast cancer (IBC) is a rare and aggressive form of breast cancer, which accounts for approximately 3% of cases of breast malignancies. Diagnosis relies largely on its clinical presentation, and despite a characteristic phenotype, underlying molecular mechanisms are poorly understood. Unique clinical presentation indicates that IBC is a distinct clinical and biological entity when compared to non-IBC. Biological understanding of non-IBC has been extrapolated into IBC and targeted therapies for HER2 positive (HER2+) and hormonal receptor positive non-IBC led to improved patient outcomes in the recent years. This manuscript reviews recent discoveries related to the underlying biology of IBC, clinical progress to date and suggests rational approaches for investigational therapies.

## INTRODUCTION

Inflammatory breast cancer (IBC) is a rare and aggressive phenotype of breast cancer encompassing approximately 3% of newly diagnosed breast tumors [1]. IBC tends to affect younger women when compared to locally advanced non-IBC with a median age at diagnosis of 57 years [2]. Among African Americans the annual incidence is estimated at 3 (2.8-3.2) per 100,000 compared to 2.1(2.1-2.2) per 100,000 among white women [3]. IBC has no histological diagnostic criteria and its diagnosis is based primarily on its clinical presentation. In 1956, Haagensen et al. described diagnostic criteria for IBC including a rapidly enlarging breast, erythema involving at least one-third of the breast, generalized induration, and biopsy proven carcinoma [4]. These remain the cornerstone diagnostic criteria for IBC [5]. IBC is also characterized by distinct skin changes including diffuse erythema and edema (*peau-d’orange*) often without a clinically evident underlying mass, which is presumed to be secondary to lymphangiogenesis and tumor emboli in the lymphatics [6–8]. It usually has an abrupt onset and rapid progression, with a high risk of axillary lymph node involvement and distant metastases at the time of initial diagnosis [9]. Despite multimodality therapy, survival rates are lower than those for non-IBC. Patients with IBC have poorer prognosis when compared to locally advanced non-IBC (5-year overall survival rates of 62% versus 81%) [10–12]. Its distinct clinical presentation and aggressive course indicate that IBC is in fact a distinct biological entity. Furthermore, the majority of newly diagnosed cases are at least stage III with approximately 30% of *de novo* stage IV [11]. Moreover, IBC has been associated with high incidence of micrometastatic disease, defined by detection of either bone marrow metastasis or circulating tumor cells (CTCs) suggesting difficulty in clinical outcome comparison with non-IBC and potential need for extended treatment beyond standard multimodality treatment [13, 14].

The standard management of IBC involves a multimodality approach, including primary systemic chemotherapy followed by mastectomy, axillary lymph node dissection, and radiation therapy, which has led to improved survival outcomes [15, 16]. The National Comprehensive Cancer Center (NCCN) guidelines list the standard approach to IBC as neoadjuvant chemotherapy with an anthracycline based regimen and a taxane [17].

HER2 is a transmembrane receptor which when overexpressed stimulates a multitude of growth factor signaling pathways in breast cancer cells [18]. IBC is associated with higher prevalence of over-expression of HER2 when compared to non-IBC with estimates ranging from 40-58% [11, 19–23]. If HER2 is overexpressed, chemotherapy with trastuzumab in combination with pertuzumab is indicated as part of the systemic preoperative regimen. Mastectomy with axillary lymph node dissection is standard in IBC patients who respond to pre-operative chemotherapy. Following surgery, postmastectomy radiation including the chest wall and supraclavicular nodes plus or minus the internal mammary nodes is recommended, as well as adjuvant endocrine therapy if indicated.

Further understanding of the molecular biology of non-IBC has led to significant advances in the treatment of breast cancer, which in conjunction with improved screening strategies, Have increased survival rates [24–26]. For instance, the comprehensive genomic analysis of breast cancer through The Cancer Genome Atlas (TCGA) program supports not only that breast cancer is a heterogeneous disease but drug development strategies should take in to account molecular aberrations specific of each subtype [27]. An increased understanding of IBC biology has been relatively hampered by its rarity and maybe more importantly by its underdiagnosis or misdiagnosis as a consequence of subjective diagnostic criteria [28]. Researchers are attempting to better characterize the molecular biology of IBC, in hopes that this will ultimately guide developmental therapeutics efforts for this rare form of breast caner. This information combined with the current understanding about the aggressive clinical presentation, high frequency of micrometastatic disease and early recurrence rates can provide leads to improved therapies. In addition, clinical trial design should also take into account the positive correlation between pathologic complete response rate (pCR) with disease-free survival benefit for a subset of patients with IBC (i.e.: HER2+ IBC) as trials assessing the efficacy of neoadjuvant treatments are inherently shorter [29]. This manuscript aims to review the discoveries in the biology of IBC while highlighting the rationale for developmental therapeutics approaches.

## GENOMIC ABERRATIONS IN IBC VS. NON-IBC

Besides differences observed from oncoprotein and gene copy number analyzes (e.g. HER2 expression/amplification) RNA next generation sequencing (NGS) technologies have allowed for better understanding of the mutational landscape of IBC [30]. A seminal work was conducted under the auspices of the IBC World Consortium by Van Laere et al. who reported results of Affymetrix gene expression profiling and molecular classification using the PAM50-algorithm derived from 137 patients with IBC and 252 patients in non-IBC [31]. Four robust IBC-sample clusters were identified, associated with the different molecular subtypes (*p* < 0.001), all of which were identified in IBC with a similar prevalence as in non-IBC, except for the luminal A subtype (19% in IBC vs. 42% in non-IBC; *p* < 0.001) and the HER2-enriched subtype (22% in IBC vs. 9% in non-IBC; *p* < 0.001). Overall, 75% of the IBC samples were classified under the classically more aggressive subtypes, basal-like, HER2-enriched, claudin-low, or luminal B subtypes, whereas these subtypes account for 54% of the non-IBC samples. The number of genes with a uniquely IBC-specific gene expression profile represented only 3% of the global expression differences. Similar results were observed when triple negative breast cancer (TNBC) IBC samples (*n*=39) were compared with TNBC, non-IBC (*n*=49) [32]. No unique IBC-specific subtypes were identified by mRNA gene-expression profiling of those tumors. Nonetheless the limited number of genes assessed by the PAM50 platform could account for failure to observe specific genomic signature in IBC samples.

Alternative techniques to gene expression assays were also implemented in order to classify IBC. Among them are epigenetic analyses such as evaluation DNA methylation pattern and also gene copy number imbalances, both of which may capture genomic signatures which account for differential tumor behavior [33, 34]. For instance, Van der Auwera et al. reported results of methylation profiling on a cohort of IBC (*n* = 19) and non-IBC (*n* = 43) samples using the Illumina Infinium platform [35]. Methylation assay comparison of IBC with non-IBC led to the identification of only four differentially methylated genes (*TJP3*, *MOGAT2*, *NTSR2* and *AGT*). Mutational landscape imbalances of 49 IBCs and 124 non-IBCs were determined using high-resolution array-comparative genomic hybridization [36]. Genomic landscapes were overall similar when comparing the two groups. Differences were however appreciated, such as the more frequent gain of 1q, 8q and 17q regions in IBCs, or the more frequent loss of 4p, 8p, 11q, and 16q regions in non-IBCs. The median percentage of probe sets displaying a copy number aberrations for a sample was numerically higher in IBCs (3.7%, range 0.01–14%) than in non-IBCs (1.9%, range 0.01–26%; *p* = 6.1), even if a great variability between samples existed for both types, suggesting that the genome of IBC is more unstable.

Most recently, Hamm et al. used a custom hybridization capture-based probe library using Agilent SureDesign portal (Agilent Technologies) to analyze 20 primary IBC specimens [37]. This panel captured full coding regions of 208 cancer relevant genes and introns of 13 genes to detect the substitution, deletions, copy number changes in the 208 targeted genes and structural rearrangements in 17 genes. Analysis of the types of genetic variants revealed that missense mutations were the most common variant (73%), followed by frameshifts (8%), splice site alterations (6%), nonsense mutations (5.5%), amplifications (5.5%), and in-frame insertions-deletions (3%). In total, NGS identified 391 genetic variants in 19 IBC tissues. The 5 most commonly altered genes were: *TP53* (58%), *HER2* (53%, all amplifications), *ATM* (53%), *APC* (37%), and *HER3* (26%).

In comparison with the mutational profile of a large non-IBC cohort (825 samples) in the TCGA, mutations in the p53 and *PIK3CA* genes are equally common when compared to the IBC mutational landscape [27]. *FGFR* and *HER2* aberrations seem to occur at similar frequency in non-IBC when compared to IBC, whereas *PIK3CA* mutations are more common in non-IBC (38%), probably because frequently associated with luminal disease. Moreover, *PTEN* abnormalities are less common in non-IBC (3%) [27, 38].

Qualitative mutational landscapes have also been performed in larger series in recent years. Ross et al. reported a comprehensive genomic profiling on 53 IBC, formalin-fixed, paraffin-embedded specimens using the hybrid capture-based, FoundationOne™ assay [39]. The 53 sequenced IBC cases harbored a total of 266 genomic alterations (GA) with an average of 5.0 GA/tumor (range 1–15). The most frequently altered genes were *TP53* (62%), *MYC* (32%), *PIK3CA* (28%), *HER2* (26%), *FGFR1* (17%), *BRCA2* (15%), and *PTEN* (15%). In the TNBC subset of IBC, 8/19 (42%) showed *MYC* amplification (median copy number 8X, range 7–20) as compared to 9/32 (28%) in non-TNBC IBC (median copy number 7X, range 6–21). Although at 32%, the *MYC* amplification in IBC appeared to represent enrichment in this tumor type, comparison with the 24% *MYC* amplification rate in the non-IBC breast cancers did not reach statistical significance (*p* = 0.26) [39]. While these small studies offer some insights in novel molecular differences, larger studies are needed to confirm and validate differences. Furthermore, non-genomic molecular characterization may be able to explain the phenotypic discordance between IBC and non-IBCs, such exploring the epigenome, metabolome, microenvironment, and immunogenic characterization.

## TRANSMEMBRANE GROWTH FACTOR RECEPTORS

### HER2

The *HER2* gene encodes a transmembrane tyrosine kinase receptor that belongs to the epithelial growth factor receptor (EGFR) family. This family of receptors includes four members (EGFR/HER1, HER2, HER3 and HER4) that function by stimulating growth factor signaling pathways such as the PI3K/AKT/mTOR pathway [18]. Activation of receptor kinase function occurs predominantly via ligand-mediated hetero- or homo-dimerization. In the case of HER2, activation is also thought to occur in a ligand-independent manner, particularly when the receptor is found to be mutated or overexpressed [40]. Targeted strategies against the HER family have been developed in the realm of breast cancer. For instance the humanized monoclonal antibodies (e.g.: trastuzumab) prevent the dimerization of HER2 with other HER receptors. Pertuzumab in particular inhibits the pairing of the most potent signaling heterodimer, HER2/HER3, thereby providing a potent strategy for dual HER2 inhibition [41]. Furthermore small molecule tyrosine kinase inhibitors such as lapatinib have the ability to inhibit the kinase activity of these HER receptors opposing further cancer cell proliferation and survival [42].

A subgroup analysis of 62 patients with HER2+ IBC enrolled in a large phase 3 trial (NOAH trial) reported a 54.8% in breast pCR to neoadjuvant trastuzumab in combination with chemotherapy, versus 19.3% for patients receiving only chemotherapy (*p* = 0.004) [43]. In this study 235 patients with HER2+ locally advanced non-IBC or IBC were randomized to treatment with or without neoadjuvant and adjuvant trastuzumab [44]. Neoadjuvant chemotherapy consisted of doxorubicin 60 mg/m^2^ plus paclitaxel 150 mg/m^2^, every 3 weeks for three cycles, followed by paclitaxel 175 mg/m^2^ administered every 3 weeks for four cycles. Cyclophosphamide (600 mg/m^2^), methotrexate (40 mg/m^2^), and fluorouracil (600 mg/m^2^) were then given on days 1 and 8 every 4 weeks for three cycles. In the overall study population chemotherapy combined with trastuzumab significantly improved in breast pCR rate to 43% and event-free survival (EFS) in patients with HER2+ breast cancer hazard ratio of 0.59, 95%CI 0.38-0.90; *p* = 0.013. After a median follow-up of 5.4 years the EFS benefit derived from trastuzumab treatment is maintained with a 5-year EFS rate of 58% (95% CI 48-66) in this patient population [45].

In the NeoSphere trial patients with HER2+ IBC were enrolled into the study but represented less than 10% of the overall study population [46]. Patients treated pertuzumab and trastuzumab plus docetaxel had a significantly improved pCR rate 45.8% [95% CI 36.1–55.7] compared with those given trastuzumab plus docetaxel; 29.0% [20.^6^–^38^.^5^]; *p* = 0.0141). Likewise in the Tryphaena phase 2 trial, among the 225 patients with HER2+ breast cancer treated with the combination of pertuzumab, trastuzumab and chemotherapy in the neoadjuvant setting only 13 had HER2+ IBC [47]. pCR rates defined as the absence of invasive carcinoma in the breast ranged from 61-66% among patients treated with dual HER2 targeted and chemotherapy.

Kaufman et al. reported results of 126 patients with relapsed or refractory HER2+ IBC who were treated with lapatinib 1500 mg once daily in a non-randomized, open-label, phase 2 study [48]. Seventy five percent of the patients had been treated with at least one line trastuzumab-based regimen. No patients achieved a complete response. The objective response rate by clinically evaluable skin-disease criteria was 40% but the objective response by rate by RECIST was only 15% (95% CI 9-24), which included only patients with metastatic or locally advanced, measurable disease. Median PFS was 14.6 weeks (95% CI 12.1–16.0), with median duration of response of 20.9 weeks (12.7–32.1). Likelihood of response to lapatinib was not affected by previous treatment with trastuzumab. A total of 45 (32%) study participants had serious adverse events, the most common ones were dyspnea (eight patients) and pleural effusion (six). Five patients had fatal adverse events that were possibly treatment related.

## EPIDERMAL GROWTH FACTOR RECEPTOR (EGFR)

EGFR is over-expressed in approximately 30% of patients with IBC and correlates with poor outcome [21, 22]. Of note EGFR has been implicated in IBC cell survival and metastasis *in vitro* and *in vivo* [49–51]. SUM149 pre-clinical cell models showed significant sensitivity to sensitivity to inhibition of EGFR and other members of the HER family [49, 52, 53]. Lapatinib is not only a potent inhibitor of the HER2 but also inhibits EGFR tyrosine kinase domains and has shown significant clinical efficacy in non-IBC HER2+ breast cancer [54, 55].

In a phase 2 single arm trial 49 patients with IBC were stratified according HER2 and EGFR expression in two different groups: cohort A (HER2+ plus or minus EGFR+ IBC) or cohort B (HER2- and EGFR+) [56]. Patients received lapatinib at 1,500 mg/d for 14 days, then lapatinib at 1,500 mg/d plus weekly paclitaxel (80 mg/m^2^) for 12 weeks, followed by surgical resection or additional chemotherapy. pCR occurred in 18.2% (95% CI, 5.2% to 40.3%) of cohort A patients. Combined clinical response rate was 78.6% (95% CI, 63.2% to 89.7%) in cohort A patients. Cohort B was terminated because of slow accrual and lack of efficacy observed in IBC patients with HER2*-*/EGFR+ tumors enrolled onto a parallel study, EGF103009 [57]. These results indicate modest activity of lapatinib in patients with HER2+ IBC. Matsuda et al. recently reported preliminary results of phase 2 trial of neoadjuvant treatment with 4 cycles of the combination of anti-EGFR monoclonal antibody panitumumab (2.5 mg/kg) combined with nab-paclitaxel (100 mg/m^2^), and carboplatin (AUC 2) weekly followed by 4 cycles of FEC (5-fluorouracil, 500 mg/m^2^; epirubicin, 100 mg/m^2^; cyclophosphamide, 500 mg/m^2^) [58]. A total of 35 patients with HER2- IBC were treated and 7 of the 16 patients with triple negative IBC achieved a pCR (44%; 95% CI: 0.20-0.70). Retrospective data support that pCR rates of < 25% are generally obtained among patients with IBC treated with and anthracycline combined a taxane in the neoadjuvant setting [59].

These results indicate that EGFR could be a valuable target for treatment development in IBC. Targeting of multiple molecular aberrations may be one the promising strategies. For instance, preliminary evidence also supports that c-MET is overexpressed in IBC [60]. Further studies are needed to evaluate the potential role of c-MET inhibition in IBC.

## mTOR/AKT PATHWAY

Aberrations in the PIK3/AKT/mTOR pathway are among the most common genomic abnormalities in breast cancer and are observed across in all subtypes of the disease [27]. In the metastatic setting mTOR inhibitor everolimus has shown significant clinical efficacy in combination with aromatase inhibitor and is now commonly used treatment option for patients with hormonal receptor, HER2- metastatic breast cancer progressing on anti-estrogen therapy [61]. *AKT* gene is not frequently mutated in breast cancer [62, 63]. However its activation by upstream molecules such as PIK3CA is a common phenomenon in breast tumors across different subtypes, which leads to activation of multiple AKT substrates controlling tumor growth and apoptosis [64, 65].

Immunohistochemical analysis of 45 cases of IBC showed over-expression of phosphorylated mTOR in approximately 90% of the cases [66]. Of note all tumor tissues were obtained from mastectomy specimens after treatment with anthracycline and or taxanes. In addition, patients with invasive, previously treated non-IBC also showed equally elevated rates of mTOR activation. Ross et al. recently reported the results of a cross-sectional study involving patients with metastatic IBC that had a diagnostic biopsy of primary tumor or metastatic lesion and subsequent NGS by Foundation One™ panel [39]. The analysis of 53 IBC tumor samples revealed a total of 266 genomic alterations were observed. Of note, abnormalities in the PI3K/AKT/mTOR pathway were seen in up 65% of the samples analyzed and 41 patients (77%) had HER2- disease. Furthermore, Hamm et al. evaluated 19 patients with primary and metastatic IBC using a targeted NGS panel that covered whole coding regions of 208 of the most common cancer related genes (copy numbers and somatic mutations) and rearrangements in 17 well characterized cancer genes [37]. Activity of the PI3K/AKT/mTOR pathway was further confirmed by immunohistochemistry for phosphorylated S6 in 95% (*n* = 18) of cases, a target of mTOR kinase activity.

A recent small cross-sectional study of 12 patients with diagnosis of HER2+ IBC who had tumor tissue biopsy upon progression on HER2 targeted therapy evaluated HER1-3 through reverse-phase protein microarrays (RPMA) assay analysis [67]. Phosphorylation of HER1-3 downstream signaling pathways such as JAK2, AKT/mTOR and MEK1/2 were analyzed. Interestingly, 83% had mTOR activation, and most of these patients also had accumulation of its downstream proteins, S6 ribosomal protein and 4E-BP-1. In addition, 78% of patients with HER2 activation also had mTOR activation indicating that the AKT/PIK3/mTOR pathway could be a mechanism of resistance to HER2 targeted therapies in IBC.

The mTORC1 inhibitor rapamycin and everolimus showed only modest inhibitory activity of IBC HER2+ and HER2- pre-clinical cell models (SUM190 and SUM149 respectively) when compared with targeted agents such as lapatinib, sorafenib, and sunitinib indicating that further understanding of the PIK3/AKT/mTOR is needed in IBC [68, 69]. In parallel, in non-IBC HER2+ tumor mTOR inhibition with everolimus combined with trastuzumab only lead to modest improvement in PFS when compared to placebo [70]. In summary, future studies are needed to investigate the potential clinical impact of the PIK3/AKT/mTOR inhibition in IBC.

## ANGIOGENESIS

The vascular endothelial growth factor (VEGF) is an important angiogenic mediator in breast cancer [71]. VEGF-A is a multifunctional cytokine widely expressed by tumor cells that acts through receptors (VEGFR-1, VEGFR-2, and neuropilin) expressed on vascular endothelium and on some other cells. It increases microvascular permeability, induces endothelial cell migration and division, reprograms gene expression, promotes endothelial cell survival, prevents senescence, and induces angiogenesis [72]. VEGF targeted therapy with the monoclonal antibody against VEGF-A, bevacizumab; seem to have modest clinical efficacy in patients with metastatic breast cancer [73, 74]. IBC is known to have high rates of endothelial cell proliferation and vascular density when compared to non-IBC, which could suggest potentially greater sensitivity to antiangiogenic therapies [75]. Wedam et al. reported results of a small cohort of 21 patients with both IBC (*n*=20) and (*n*=1) non-IBC treated with bevacizumab for cycle 1 (15 mg/kg on day 1) followed by six cycles of bevacizumab with doxorubicin (50 mg/m^2^) and docetaxel (75 mg/m^2^) every 3 weeks in the neoadjuvant setting [76]. Tumor biopsies were collected on cycles 1, 4, and 7; a median decrease of 66.7% in phosphorylated VEGFR2 in tumor cells and median increase of 128.9% in tumor apoptosis were seen after bevacizumab alone. These changes persisted with the addition of chemotherapy. One patient had a complete pathological had a complete pCR and 14 have partial clinical response overall response rate (ORR) of 67% (95% CI, 43% to 85.4%). The ORR results are comparable with historical controls treated with anthracycline/taxane combination without bevacizumab in the neoadjuvant setting (ORR 81%) [77].

The BEVERLY-1 (UCBG-0802) trial was a phase 2, single-arm trial, in which women with non-metastatic HER2- IBC were treated with neoadjuvant intravenous fluorouracil (500 mg/m^2^), epirubicin (100 mg/m^2^), cyclophosphamide (500 mg/m^2^), and bevacizumab (15 mg/kg) during cycles 1–4 (21 days-cycle), then docetaxel (100 mg/m^2^) and bevacizumab during cycles 5–8 [78]. After surgery, patients received adjuvant intravenous bevacizumab. After neoadjuvant therapy, only 19 of the 100 patients evaluable for efficacy analysis achieved a pCR. The most frequent grade 3–4 events during the neoadjuvant phase were neutropenia (89%), febrile neutropenia (37%), and mucositis 23%) and during the adjuvant phase the most frequent grade 3–4 adverse event was proteinuria (7%). One (1%) patient died of thrombotic microangiopathy after cycle 1, which was thought to be related to bevacizumab. Two patients (3%) developed transitory heart failure.

In the BEVERLY-2 study 52 patients with HER2+ IBC were treated with fluorouracil, epirubicin, cyclophosphamide, and bevacizumab (cycles 1–4) and docetaxel, trastuzumab, and bevacizumab (cycles 5–8) before surgery, followed by trastuzumab and bevacizumab for 30 weeks after surgery [80]. After neoadjuvant therapy, 33 of 52 patients had a pCR according to central review (63.5%, 95% CI 49.4–77.5). The most common adverse events were asthenia and nausea (both occurred in 36 [69%] of 52 patients). Only one grade 3 or worse adverse event regarded as related to bevacizumab was reported (hypertension, one patient). It is important to note that the pCR rates of this HER2+ IBC cohort was comparable to previously published studies, which did not add bevacizumab to the neoadjuvant treatment regimen of patient IBC [81]. Taken together these results indicate that the addition of bevacizumab to chemotherapy does not significantly improve pathological complete response rate in IBC.

One could hypothesize that higher expression of several non-VEGF angiogenic, lymphangiogenic, and vasculogenic factors could make blockade of VEGF by bevacizumab insufficient [7]. Indeed, pre-clinical data support that multityrosine kinase inhibitors such as sorafenib and sunitinib exert inhibitory activity against SUM149 IBC cell models [68]. For instance, pazopanib is an oral angiogenesis inhibitor targeting VEGF receptors 1-3, platelet-derived growth factor receptors-α/−β [82, 83]. Cristofanilli et al. reported results of a multi-center phase 2 study evaluating lapatinib, pazopanib, or the combination in patients with relapsed HER2+ inflammatory breast cancer [84]. In Cohort 1, 76 patients were randomized 1:1 to receive lapatinib 1,500 mg plus placebo or lapatinib 1,500 mg plus pazopanib 800 mg (double-blind) once daily until disease progression, unacceptable toxicity, or death. Due to high-grade diarrhea observed with this dose combination in another study (VEG20007), Cohort 1 was closed. The protocol was amended such that an additional 88 patients (Cohort 2) were randomized in a 5:5:2 ratio to receive daily monotherapy lapatinib 1,500 mg, lapatinib 1,000 mg plus pazopanib 400 mg, or pazopanib 800 mg monotherapy, respectively. The primary endpoint was ORR and secondary endpoints included duration of response, progression-free survival (PFS), overall survival, and safety. In Cohort 1, ORR for the lapatinib (*n* = 38) and combination (*n* = 38) arms was 29% and 45%, respectively; median PFS was 16.1 and 14.3 weeks, respectively. Grade 3 adverse events were more frequent in the combination arm (71%) than in the lapatinib arm (24%). Dose reductions and interruptions due to AEs were also more frequent in the combination arm (45 and 53%, respectively) than in the lapatinib monotherapy arm (0 and 11%, respectively). In Cohort 2, ORR for patients treated with lapatinib (*n* = 36), lapatinib plus pazopanib (*n* = 38), and pazopanib (*n* = 13) was 47, 58, and 31%, respectively; median PFS was 16.0, 16.0, and 11.4 weeks, respectively. In the lapatinib, combination, and pazopanib therapy arms, grade ≥3 AEs were reported for 17, 50, and 46% of patients, respectively. The lapatinib-pazopanib combination was associated with a numerically higher ORR but no increase in PFS compared to lapatinib alone, which was a secondary endpoint of the study. The combination also had increased toxicity resulting in more dose reductions, modifications, and treatment delays. As novel agents are developed that are better tolerated, the improve response rates in this study may suggest a role for multi-target angiogenesis agents in IBC.

## JANUS KINASE/SIGNAL TRANSDUCER AND ACTIVATOR OF TRANSCRIPTION (JAK/STAT) PATHWAY

JAK kinases are activated through tyrosine phosphorylation of the cytoplasmic domains of cytokine receptors upon cytokine binding. JAK2 activation promotes recruitment to the receptor complex of the transcription factors STAT3 and STAT5 [85]. JAK2-mediated STAT phosphorylation leads to the formation of stable homodimers and heterodimers, which leads to their nuclear translocation. Once in the nucleus, STAT molecules bind specific promoter DNA sequences that result in the transcription of genes that regulate cell proliferation, differentiation, and apoptosis (e.g., Bcl-xL, cyclin D1, and PIM1) [85, 86]. STAT3 has been implicated in many aspects of tumorigenesis, including differentiation, proliferation, apoptosis, increased sensitivity to cytotoxic agents, angiogenesis, recruitment of immune cells, and metastasis [87]. Evidence supports that, in breast cancer, JAK2 activates STAT3 and is found to be significantly activated when compared to non-neoplastic breast tissue [88, 89]. In one series of 45 samples of previously treated IBC samples immunohistochemical analysis showed activated JAK2 (pJAK2) levels were similar between IBC (95.2%, 1+ or 2+) and treated IDC (91.7%, 1+ or 2+; 4.2%, 3+), untreated IDC had lower levels (80.0%, 0; 20.0%, 1+) (*p* < 0.0001). For pSTAT3, 55.0% of IBC tumors had 1+ or 2+ levels with 45% of tumors having level 0. In treated IDC, 62.5% had 1+ or 2+ expression with 37.5% of tumors having level 0. This is in contrast to untreated IDC where 92.3% of tumors had 0 level (*p* = 0.0001) [66]. These results indicate that JAK/STAT pathway activation could lead to treatment resistance in IBC. A phase 1/2 study of combination ruxolitinib (kinase inhibitor of JAK/STAT) with neoadjuvant chemotherapy for triple negative IBC is ongoing (NCT02041429).

## CELL CYCLE CONTROL PATHWAYS

**Figure 1:**
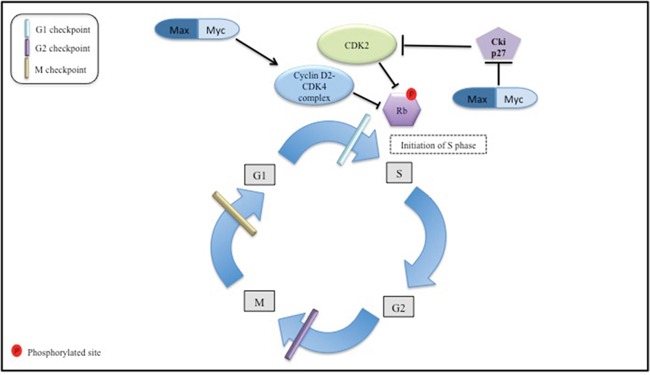
The Myc interaction with the cell cycle machinery this figure is a simplified depiction of the role Myc oncoprotein in regulation of the cell cycle machinery. The normal cell replication processes are represented i.e., G1 first growth period to S DNA replication phase, G2 second growth period, and M, which is the mitosis period. Cyclin D2-CDK4 and CDK2 inactivate retinoblastoma protein (Rb) through phosphorylation. The latter event allows for cell cycle progression from G1 to S phase. Max/Myc complex targets cyclinD2-CDK4 complex formation ultimately stimulating cell cycle progression. Also cycle dependent kinase 2 (CDK2) complex activation is depicted through the abrogation of inhibitory action of Cki27 by active Max/Myc complex. When active CDK2 complex promotes initiation of S phase.

MYC oncoprotein interacts closely with the cell cycle machinery in depriving the cell of the normal control of progression through the G_1_ phase of the cell cycle into the S phase. MYC protein acting with its Max partner is able to induce expression of the growth-promoting cyclin D2 and CDK4 (Figure 1) [90, 91]. At the same time MYC can promote degradation of p27^Kip1^ CDK inhibitor as well as E2F1 favoring advancement into the S phase [92, 93].

Ross et al. reported the results of genomic sequencing of 53 IBC samples from the primary or a metastatic site [94]. Alterations in cell cycle regulatory genes were identified as follows: *MYC* (31%), *CCND1* (9%), *RB1* alterations (9%), and *CDKN2A* (8%). All *MYC* aberrations consisted of amplifications. Of the 19 TNBC IBC cases 8 (42%) had *MYC* amplification, whereas only 9 among 36 non-TNBC IBC cases harbored *MYC* amplification in 9 (25%). Indeed, the luminal, estrogen receptor- positive, IBC cell line (SUM190) exhibited significant sensitivity to CDK4/6 inhibitor (palbociclib) when compared to other solid tumors [95]. While a standard of care in conjunction with anti-hormone therapy in ER+ MBC, the clinical efficacy of palbociclib has not been reported in IBC.

## RHOC GTPASE

In a previous series of studies, RhoC GTPase overexpression has been identified in >90% of IBCs and defined *RhoC* as a mammary oncogene involved in conferring the metastatic phenotype in IBC and estrogen receptor negative non-IBC [96–98]. Protein farnesyl transferase inhibitors revert the RhoC GTPase-induced inflammatory breast cancer phenotype [99]. Tipifarnib is an oral protein farnesyl transferase inhibitor. Despite the pre-clinical rationale, in a phase 2 trial of 22 patients with HER2- IBC, only one patient achieved a pCR after treatment with tipifarnib and conventional anthracycline/taxane-based neoadjuvant chemotherapy indicating lack of single activity of tipifarnib [100]. One potential strategy for future drug development could be to combine farnesyltranferase enzyme inhibitors with STAT3 targeted therapies in IBC and there also seems to be interaction between these two pathways in non-IBC [101]. Furthermore, IBC pre-clinical models support that RhoC GTPase is a substrate for Akt1 and its phosphorylation is absolutely essential for IBC cell invasion [102]. Future drug development should take into account potential interactions between RhoC GTPase and the PIK3/AKT/mTOR pathway.

## ANAPLASTIC LYMPHOMA KINASE (ALK)

*EML4/ALK* fusion is present in about 5% of patients with non-small cell lung cancer (NSCLC) [103]. The presence of EML4/ALK translocation is predictive of clinically meaningful benefit from treatment with the oral tyrosine kinase inhibitor crizotinib and alectinib in patients with NSCLC [104, 105]; ALK aberrations are also thought be rare in non-IBC (<1%) [106]. Analysis of 25 samples of IBC for ALK genetic abnormalities was performed. These studies revealed that 20/25 (80%) had some form of ALK aberration (i.e.: increased copy number, low level ALK gene amplification, or ALK gene expression), with an increased prevalence of ALK alterations in basal-like IBC. One of 25 patients was identified as having an *EML4-ALK* translocation [107]. A recent preliminary report indicated that ALK protein over-expression is not thought to be a feature of IBC [108]. The predictive value of *ALK* genomic aberrations is being assessed in an ongoing phase 1 trial in which patients with solid tumors including IBC, will be treated with LDK378 (selective inhibitor of ALK) (NCT01283516).

## EPIGENETIC MODULATION

Polycomb group (PcG) protein enhancer of Zeste homolog 2 (EZH2) is among the DNA methylation modulation mechanisms, which interacts-within the context of the PcG repressive complexes 2 and 3 (PRC2/3)-with DNA methyltransferases (DNMTs) and associates with DNMT activity *in vivo*. Binding of DNMTs to several EZH2-repressed genes depends on the presence of EZH2 [109]. EZH2 is frequently expressed (~75%) in human IBC and its expression correlates with worse clinical outcome [110]. EZH2 is expressed at higher levels in human IBC cell lines compared with normal human mammary epithelial cells, and the knockdown of EZH2 expression significantly suppressed cell growth and tumor spheroid formation of human IBC cells *in vitro*. In addition, EZH2 knockdown inhibited the migration and invasion of IBC cells. Significantly, EZH2 knockdown suppressed the angiogenesis and tumor growth of IBC cells *in vivo* [111]. Furthermore pre-clinical evidence supports that epigenic modulation through histone deacetylase inhibitors (HDAC) may be promising in IBC models. Robertson et al. reported results of *in vivo* and *in vitro* IBC models supporting antitumor activity of the HDAC inhibitor romidepsin [112]. Not only did HDAC inhibition cause lower levels of VEGF-A and hypoxia-induced factor 1α in a xenograft model but it also blocked self-renewal/clonogenicity of tumor spheroids. In addition, romidepsin alone effectively inhibited SUM149 primary tumor growth and was synergistic with paclitaxel in blocking development of SUM149 metastatic lesions at multiple sites. Preliminary results from a phase 1 trial which tested the combination of romidepsin with nab-paclitaxel among 9 patients with metastatic, refractory HER2- IBC showed one complete response and two disease stabilizations [113]. Toxicities related to romidepsin included neutropenia, anemia and fatigue. Ongoing studies are evaluating HDAC inhibitors alone or in combination with targeted therapies [114, 115].

## IMMUNE CHECKPOINT INHIBITORS

Immunotherapy with checkpoint inhibitors has made a significant impact in the treatment of melanoma, renal cell carcinoma and NSCLC in recent years [116–119]. New agents such as nivolumab and pembrolizumab [a fully human IgG4 programmed death 1 (PD-1) immune-checkpoint inhibitor antibody] selectively blocks the interaction of the PD-1 receptor with its two known programmed death ligands, PD-L1 and PD-L2, disrupting the negative signal that regulates T-cell activation and proliferation [120]. There is preliminary evidence of positive correlation between high mutational burden of tumors and clinical benefit from immunotherapy strategies (i.e. checkpoint inhibitors anti-CTLA-4 and anti-PD-1 antibodies), with remarkable effects seen with tumors displaying the highest rates of mutations such as melanoma [121, 122]. This is also illustrated by the anti-tumoral immunologic response to anti-PD-1 antibody in patients with colorectal cancer and increased mutational burden secondary to mismatch repair deficiency [123]. Nonetheless lack of definition and standardization of measures of tumor mutational load, and prospective validation of its predictive value indicate that better understanding of biomarkers predictive of benefit from immune checkpoint inhibition remains an unmet need.

The potential importance of immune checkpoint-guided therapy in breast cancer is underscored by recent report of PD-1 inhibitor activity in triple negative non-IBC. Pembrolizumab, which is a monoclonal anti-PD-1 antibody was tested in a phase 1b trial on 32 female patients with PD-L1 immunohistochemistry (IHC) + and heavily pretreated metastatic recurrent triple negative non-IBC. The disease control rate (i.e., percentage of patients with best response of complete response, partial response, or stable disease for ≥ 24 weeks) was 25.9% (95% CI, 11.1% to 46.3%) [124]. Avelumab, an anti-PD-L1 IgG1 antibody, showed modest anti-tumor activity among 57 patients with triple negative non-IBC with only 5 partial responses observed (8.8%; 95% CI: 2.9, 19.3) [125]. In patients with triple negative non-IBC who had PD-L1+ immune cells within the tumor, 44.4% (4 of 9) had partial responses, compared with 2.6% (1 of 39) for triple negative non-IBC and PD-L1– immune cells.

The role of immune infiltrate and immune checkpoints was also investigated in relation with genomic abnormalities in IBC samples [37]. The pathological examination of 20 IBC tissue samples identified a subset of IBC tumors associated with infiltration of immune cells. IHC staining identified the majority of infiltrating cell populations as CD8+ cytotoxic T cells and high levels of CD8+ infiltration were observed in 5/12 tumors. In order to explore the possible role of PD-L1 in IBC, the investigators performed IHC staining of IBC tissues. Evaluation of PD-L1 staining demonstrated low-intensity tumor cell staining in 3/12 tumors studied and high-intensity tumor cell staining in 1/12 tumors. PD-L1 mRNA expression has been reported to by as high as 38% among patients with IBC, which is higher that non-IBC (28%) and correlates positively with pCR [126].

Notably, somatic mutation rates were significantly higher in high infiltration *vs*. low infiltration tumors (*p*<0.05) [37]. The authors speculated that this correlation between somatic mutation rate and immune cell infiltration might be related to the exposure of tumor neo-antigens to the immune system. A phase 2 clinical trial for patients with metastatic IBC assessing the efficacy of anti-PD-1 inhibitor monoclonal antibody (pembrolizumab) is under development (NCT02411656) (Table 1).

**Table 1:** Drugs under development for IBC – trials accrue patients with IBC only

Agent (s)	IBC subtype	Phase of study	Mechanism of action targeted therapy	Clinicaltrial.gov Identification number
Ruxolitinib	TN	2	JAK/STAT TKI	NCT02041429
Paclitaxel/trastuzumab/pertuzumab	HER2 +	2	Anti-HER2 mAb	NCT01796197
Afatinib	HER2 +	2	EGFR and HER2 TKI	NCT01325428
Nintedanib	HER2 -	2	Multi TKI	NCT02389764
Dovitinib	HER2 -	2	Multi TKI	NCT01262027
Panitumumab/nab-paclitaxel/carboplatin	HER2 -	2	Anti-EGFR mAb	NCT01036087
Eribulin/doxorubicin/cyclophosphamide	HER2 -	2	–	NCT02623972
Pembrolizumab	NS	2	Anti-PD-1 mAb	NCT02411656
SU5416/doxorubicin	NS	1	VEGFR TKI	NCT00005822
Nab-paclitaxel/gemcitabine/epirubicin	NS	2	–	NCT00193206
Bevacizumab/cyclophosphamide/ 5-FU/epirubicin	NS	1	Anti-VEGF mAb	NCT01880385
Bevacizumab	NS	2	Anti-VEGF mAb	NCT00016549
Docetaxel/5-FU	NS	3	–	NCT02324088

Accessed on July 18^th^ 2016 at www.clinicaltrials.gov

Abbreviations: Epithelial growth factor receptor (EGFR), fluorouracil (5-FU), inflammatory breast cancer (IBC), janus kinase/ signal transducers and activators of transcription (JAK/STAT), monoclonal anti-body (mAb), receptor tyrosine-protein kinase erbB-2 (HER2), triple negative (TN), tyrosine kinase inhibitor (TKI), vascular endothelial growth factor (VEGF), vascular endothelial growth factor receptor (VEGFR).

## DISCUSSION

IBC is rare but aggressive disease, in which improvements in therapeutic strategies are urgently needed. Its presentation, aggressive clinical course, and frequent distant recurrence indicate that IBC is in fact a distinct clinical biological entity rather than a subtype on the spectrum of locally advanced breast cancer. Key molecular differences include significant alterations in the PI3K and JAK/STAT pathways, elevated aberrations in DNA-repair genes and cell-cycle regulations suggesting of significant genomic instability contributing to treatment resistance. Furthermore higher rates of HER2 overexpression/amplification are seen in IBC and neoadjuvant treatment with trastuzumab resulted in improved outcomes for patients with HER2+ IBC [43]. There is no prospective trial appropriately powered to assess the efficacy of neoadjuvant treatment with chemotherapy combined with pertuzumab and trastuzumab in HER2+ IBC. Nonetheless based on improved pCR rates seen in non-IBC these agents are recommended by current guidelines for the neoadjuvant treatment of HER2+ IBC.

As with other rare or under diagnosed diseases the evidence for the treatment of IBC stems from small prospective trials and subgroup analysis of larger non-IBC prospective studies. As IBC has no histological diagnostic criteria, every effort must be made to accomplish international standardization of diagnosis and treatment and facilitate future research [5, 127]. Research groups have also initiated prospective biorepository studies in order to facilitate future tumor tissue and blood-based biomarker studies in IBC (NCT00477100, NCT00646555, NCT00340158). Efforts are increasing to improve the biological understanding of IBC and to conduct clinical trials, which are specific for patients with IBC (Table 1). Nonetheless in the ever-expanding field of biomarker-based research reliable correlations between pCR and tumor genomic aberrations remain to be determined in IBC, except for HER2 amplification [128].

Further understanding of molecular biology of IBC focusing on the tumor microenvironment and immunity may help explain the different clinical behaviors of IBC and non-IBC e.g. the role of mesenchymal transitional cells in promoting E-cadherin (pivotal to IBC metastasis) expression in IBC cell models and metastases in xenografts [50, 75].

## References

[1] Levine PH, Veneroso C (2008). The epidemiology of inflammatory breast cancer. Semin Oncol.

[2] Anderson WF, Schairer C, Chen BE, Hance KW, Levine PH (2005). Epidemiology of inflammatory breast cancer (IBC). Breast Dis.

[3] Goldner B, Behrendt CE, Schoellhammer HF, Lee B, Chen SL (2014). Incidence of inflammatory breast cancer in women, 1992-2009, United States. Ann Surg Oncol.

[4] Haagensen CD (1956). Diseases of the female breast. Trans N Engl Obstet Gynecol Soc.

[5] Dawood S, Merajver SD, Viens P, Vermeulen PB, Swain SM, Buchholz TA, Dirix LY, Levine PH, Lucci A, Krishnamurthy S, Robertson FM, Woodward WA, Yang WT (2011). International expert panel on inflammatory breast cancer: consensus statement for standardized diagnosis and treatment. Ann Oncol.

[6] Walshe JM, Swain SM (2005). Clinical aspects of inflammatory breast cancer. Breast Dis.

[7] Van der Auwera I, Van Laere SJ, Van den Eynden GG, Benoy I, van Dam P, Colpaert CG, Fox SB, Turley H, Harris AL, Van Marck EA, Vermeulen PB, Dirix LY (2004). Increased angiogenesis and lymphangiogenesis in inflammatory versus noninflammatory breast cancer by real-time reverse transcriptase-PCR gene expression quantification. Clin Cancer Res.

[8] Alpaugh ML, Tomlinson JS, Kasraeian S, Barsky SH (2002). Cooperative role of E-cadherin and sialyl-Lewis X/A-deficient MUC1 in the passive dissemination of tumor emboli in inflammatory breast carcinoma. Oncogene.

[9] Jaiyesimi IA, Buzdar AU, Hortobagyi G (1992). Inflammatory breast cancer: a review. J Clin Oncol.

[10] Hance KW, Anderson WF, Devesa SS, Young HA, Levine PH (2005). Trends in inflammatory breast carcinoma incidence and survival: the surveillance, epidemiology, and end results program at the National Cancer Institute. J Natl Cancer Inst.

[11] Matro JM, Li T, Cristofanilli M, Hughes ME, Ottesen RA, Weeks JC, Wong YN (2015). Inflammatory breast cancer management in the national comprehensive cancer network: the disease, recurrence pattern, and outcome. Clin Breast Cancer.

[12] Budd GT, Barlow WE, Moore HC, Hobday TJ, Stewart JA, Isaacs C, Salim M, Cho JK, Rinn KJ, Albain KS, Chew HK, Burton GV, Moore TD (2015). SWOG S0221: a phase III trial comparing chemotherapy schedules in high-risk early-stage breast cancer. J Clin Oncol.

[13] Braun S, Vogl FD, Naume B, Janni W, Osborne MP, Coombes RC, Schlimok G, Diel IJ, Gerber B, Gebauer G, Pierga JY, Marth C, Oruzio D (2005). A pooled analysis of bone marrow micrometastasis in breast cancer. N Engl J Med.

[14] Janni WJ, Rack B, Terstappen LW, Pierga JY, Taran FA, Fehm T, Hall C, de Groot MR, Bidard FC, Friedl TW, Fasching PA, Brucker SY, Pantel K (2016). Pooled Analysis of the Prognostic Relevance of Circulating Tumor Cells in Primary Breast Cancer. Clin Cancer Res.

[15] Panades M, Olivotto IA, Speers CH, Shenkier T, Olivotto TA, Weir L, Allan SJ, Truong PT (2005). Evolving treatment strategies for inflammatory breast cancer: a population-based survival analysis. J Clin Oncol.

[16] Dawood S, Ueno NT, Valero V, Woodward WA, Buchholz TA, Hortobagyi GN, Gonzalez-Angulo AM, Cristofanilli M (2012). Identifying factors that impact survival among women with inflammatory breast cancer. Ann Oncol.

[17] Guidelines N NCCN Clinical Practical Guidelines in Oncology (NCCN Guidelines) Breast Cancer Version 3.215.

[18] Gajria D, Chandarlapaty S (2011). HER2-amplified breast cancer: mechanisms of trastuzumab resistance and novel targeted therapies. Expert Rev Anticancer Ther.

[19] Parton M, Dowsett M, Ashley S, Hills M, Lowe F, Smith IE (2004). High incidence of HER-2 positivity in inflammatory breast cancer. Breast.

[20] Sawaki M, Ito Y, Akiyama F, Tokudome N, Horii R, Mizunuma N, Takahashi S, Horikoshi N, Imai T, Nakao A, Kasumi F, Sakamoto G, Hatake K (2006). High prevalence of HER-2/neu and p53 overexpression in inflammatory breast cancer. Breast Cancer.

[21] Cabioglu N, Gong Y, Islam R, Broglio KR, Sneige N, Sahin A, Gonzalez-Angulo AM, Morandi P, Bucana C, Hortobagyi GN, Cristofanilli M (2007). Expression of growth factor and chemokine receptors: new insights in the biology of inflammatory breast cancer. Ann Oncol.

[22] Charafe-Jauffret E, Tarpin C, Bardou VJ, Bertucci F, Ginestier C, Braud AC, Puig B, Geneix J, Hassoun J, Birnbaum D, Jacquemier J, Viens P (2004). Immunophenotypic analysis of inflammatory breast cancers: identification of an 'inflammatory signature'. J Pathol.

[23] Guy PM, Platko JV, Cantley LC, Cerione RA, Carraway KL (1994). Insect cell-expressed p180erbB3 possesses an impaired tyrosine kinase activity. Proc Natl Acad Sci U S A.

[24] Turner NC, Ro J, Andre F, Loi S, Verma S, Iwata H, Harbeck N, Loibl S, C Huang Bartlett, Zhang K, Giorgetti C, Randolph S, Koehler M (2015). Palbociclib in Hormone-Receptor-Positive Advanced Breast Cancer. N Engl J Med.

[25] Verma S, Miles D, Gianni L, Krop IE, Welslau M, Baselga J, Pegram M, Oh DY, Dieras V, Guardino E, Fang L, Lu MW, Olsen S (2012). Trastuzumab emtansine for HER2-positive advanced breast cancer. N Engl J Med.

[26] Baselga J, Cortes J, Kim SB, Im SA, Hegg R, Im YH, Roman L, Pedrini JL, Pienkowski T, Knott A, Clark E, Benyunes MC, Ross G (2012). Pertuzumab plus trastuzumab plus docetaxel for metastatic breast cancer. N Engl J Med.

[27] Cancer Genome Atlas N (2012). Comprehensive molecular portraits of human breast tumours. Nature.

[28] Kim T, Lau J, Erban J (2006). Lack of uniform diagnostic criteria for inflammatory breast cancer limits interpretation of treatment outcomes: a systematic review. Clin Breast Cancer.

[29] Pierga JY, Petit T, Levy C, Ferrero JM, Campone M, Gligorov J, Lerebours F, Roche H, Bachelot T, Charafe-Jauffret E, Bonneterre J, Hernandez J, Bidard FC (2015). Pathological response and circulating tumor cell count identifies treated HER2+ inflammatory breast cancer patients with excellent prognosis: BEVERLY-2 survival data. Clin Cancer Res.

[30] Carneiro BA, Costa R, Taxter T, Chandra S, Chae YK, Cristofanilli M, Giles FJ (2016). Is Personalized Medicine Here? Oncology (Williston Park).

[31] Van Laere SJ, Ueno NT, Finetti P, Vermeulen P, Lucci A, Robertson FM, Marsan M, Iwamoto T, Krishnamurthy S, Masuda H, van Dam P, Woodward WA, Viens P (2013). Uncovering the molecular secrets of inflammatory breast cancer biology: an integrated analysis of three distinct affymetrix gene expression datasets. Clin Cancer Res.

[32] Masuda H, Baggerly KA, Wang Y, Iwamoto T, Brewer T, Pusztai L, Kai K, Kogawa T, Finetti P, Birnbaum D, Dirix L, Woodward WA, Reuben JM (2013). Comparison of molecular subtype distribution in triple-negative inflammatory and non-inflammatory breast cancers. Breast Cancer Res.

[33] Bae YK, Brown A, Garrett E, Bornman D, Fackler MJ, Sukumar S, Herman JG, Gabrielson E (2004). Hypermethylation in histologically distinct classes of breast cancer. Clin Cancer Res.

[34] Loo LW, Grove DI, Williams EM, Neal CL, Cousens LA, Schubert EL, Holcomb IN, Massa HF, Glogovac J, Li CI, Malone KE, Daling JR, Delrow JJ (2004). Array comparative genomic hybridization analysis of genomic alterations in breast cancer subtypes. Cancer Res.

[35] Van der Auwera I, Yu W, Suo L, Van Neste L, van Dam P, Van Marck EA, Pauwels P, Vermeulen PB, Dirix LY, Van Laere SJ (2010). Array-based DNA methylation profiling for breast cancer subtype discrimination. PLoS One.

[36] Bekhouche I, Finetti P, Adelaide J, Ferrari A, Tarpin C, Charafe-Jauffret E, Charpin C, Houvenaeghel G, Jacquemier J, Bidaut G, Birnbaum D, Viens P, Chaffanet M (2011). High-resolution comparative genomic hybridization of inflammatory breast cancer and identification of candidate genes. PLoS One.

[37] Hamm CA, Moran D, Rao K, Trusk PB, Pry K, Sausen M, Jones S, Velculescu VE, Cristofanilli M, Bacus S (2016). Genomic and Immunological Tumor Profiling Identifies Targetable Pathways and Extensive CD8+/PDL1+ Immune Infiltration in Inflammatory Breast Cancer Tumors. Mol Cancer Ther.

[38] Helsten T, Elkin S, Arthur E, Tomson BN, Carter J, Kurzrock R (2016). The FGFR Landscape in Cancer: Analysis of 4,853 Tumors by Next-Generation Sequencing. Clin Cancer Res.

[39] Ross JS, Ali SM, Wang K, Khaira D, Palma NA, Chmielecki J, Palmer GA, Morosini D, Elvin JA, Fernandez SV, Miller VA, Stephens PJ, Cristofanilli M (2015). Comprehensive genomic profiling of inflammatory breast cancer cases reveals a high frequency of clinically relevant genomic alterations. Breast Cancer Res Treat.

[40] Yarden Y (2001). The EGFR family and its ligands in human cancer. signalling mechanisms and therapeutic opportunities. Eur J Cancer.

[41] Harbeck N, Beckmann MW, Rody A, Schneeweiss A, Muller V, Fehm T, Marschner N, Gluz O, Schrader I, Heinrich G, Untch M, Jackisch C (2013). HER2 Dimerization Inhibitor Pertuzumab - Mode of Action and Clinical Data in Breast Cancer. Breast Care (Basel).

[42] Burris HA, HI 3rd Hurwitz, Dees EC, Dowlati A, Blackwell KL, O'Neil B, Marcom PK, Ellis MJ, Overmoyer B, Jones SF, Harris JL, Smith DA, Koch KM (2005). Phase I safety, pharmacokinetics, and clinical activity study of lapatinib (GW572016), a reversible dual inhibitor of epidermal growth factor receptor tyrosine kinases, in heavily pretreated patients with metastatic carcinomas. J Clin Oncol.

[43] Baselga J SV, Manikhas GM (2007). Efficacy of neoadjuvant trastuzumab in patients with inflammatory breast cancer: data from the NOAH (Neoadjuvant Herceptin) phase III trial. Eur J Cancer Suppl.

[44] Gianni L, Eiermann W, Semiglazov V, Manikhas A, Lluch A, Tjulandin S, Zambetti M, Vazquez F, Byakhow M, Lichinitser M, Climent MA, Ciruelos E, Ojeda B (2010). Neoadjuvant chemotherapy with trastuzumab followed by adjuvant trastuzumab versus neoadjuvant chemotherapy alone, in patients with HER2-positive locally advanced breast cancer (the NOAH trial): a randomised controlled superiority trial with a parallel HER2-negative cohort. Lancet.

[45] Gianni L, Eiermann W, Semiglazov V, Lluch A, Tjulandin S, Zambetti M, Moliterni A, Vazquez F, Byakhov MJ, Lichinitser M, Climent MA, Ciruelos E, Ojeda B (2014). Neoadjuvant and adjuvant trastuzumab in patients with HER2-positive locally advanced breast cancer (NOAH): follow-up of a randomised controlled superiority trial with a parallel HER2-negative cohort. Lancet Oncol.

[46] Gianni L, Pienkowski T, Im YH, Roman L, Tseng LM, Liu MC, Lluch A, Staroslawska E, J de la Haba-Rodriguez, Im SA, Pedrini JL, Poirier B, Morandi P (2012). Efficacy and safety of neoadjuvant pertuzumab and trastuzumab in women with locally advanced, inflammatory, or early HER2-positive breast cancer (NeoSphere): a randomised multicentre, open-label, phase 2 trial. Lancet Oncol.

[47] Schneeweiss A, Chia S, Hickish T, Harvey V, Eniu A, Hegg R, Tausch C, Seo JH, Tsai YF, Ratnayake J, McNally V, Ross G, Cortes J (2013). Pertuzumab plus trastuzumab in combination with standard neoadjuvant anthracycline-containing and anthracycline-free chemotherapy regimens in patients with HER2-positive early breast cancer: a randomized phase II cardiac safety study (TRYPHAENA). Ann Oncol.

[48] Kaufman B, Trudeau M, Awada A, Blackwell K, Bachelot T, Salazar V, DeSilvio M, Westlund R, Zaks T, Spector N, Johnston S (2009). Lapatinib monotherapy in patients with HER2-overexpressing relapsed or refractory inflammatory breast cancer: final results and survival of the expanded HER2+ cohort in EGF103009, a phase II study. Lancet Oncol.

[49] Mu Z, Klinowska T, Dong X, Foster E, Womack C, Fernandez SV, Cristofanilli M (2014). AZD8931, an equipotent, reversible inhibitor of signaling by epidermal growth factor receptor (EGFR), HER2, and HER3: preclinical activity in HER2 non-amplified inflammatory breast cancer models. J Exp Clin Cancer Res.

[50] Lacerda L, Debeb BG, Smith D, Larson R, Solley T, Xu W, Krishnamurthy S, Gong Y, Levy LB, Buchholz T, Ueno NT, Klopp A, Woodward WA (2015). Mesenchymal stem cells mediate the clinical phenotype of inflammatory breast cancer in a preclinical model. Breast Cancer Res.

[51] Buchheit CL, Angarola BL, Steiner A, Weigel KJ, Schafer ZT (2015). Anoikis evasion in inflammatory breast cancer cells is mediated by Bim-EL sequestration. Cell Death Differ.

[52] Mueller KL, Yang ZQ, Haddad R, Ethier SP, Boerner JL (2010). EGFR/Met association regulates EGFR TKI resistance in breast cancer. J Mol Signal.

[53] Baillo A, Giroux C, Ethier SP (2011). Knock-down of amphiregulin inhibits cellular invasion in inflammatory breast cancer. J Cell Physiol.

[54] Rusnak DW, Lackey K, Affleck K, Wood ER, Alligood KJ, Rhodes N, Keith BR, Murray DM, Knight WB, Mullin RJ, Gilmer TM (2001). The effects of the novel, reversible epidermal growth factor receptor/ErbB-2 tyrosine kinase inhibitor, GW2016, on the growth of human normal and tumor-derived cell lines in vitro and in vivo. Mol Cancer Ther.

[55] Geyer CE, Forster J, Lindquist D, Chan S, Romieu CG, Pienkowski T, Jagiello-Gruszfeld A, Crown J, Chan A, Kaufman B, Skarlos D, Campone M, Davidson N (2006). Lapatinib plus capecitabine for HER2-positive advanced breast cancer. N Engl J Med.

[56] Boussen H, Cristofanilli M, Zaks T, DeSilvio M, Salazar V, Spector N (2010). Phase II study to evaluate the efficacy and safety of neoadjuvant lapatinib plus paclitaxel in patients with inflammatory breast cancer. J Clin Oncol.

[57] Johnston S, Trudeau M, Kaufman B, Boussen H, Blackwell K, LoRusso P, Lombardi DP, S Ben Ahmed, Citrin DL, DeSilvio ML, Harris J, Westlund RE, Salazar V (2008). Phase II study of predictive biomarker profiles for response targeting human epidermal growth factor receptor 2 (HER-2) in advanced inflammatory breast cancer with lapatinib monotherapy. J Clin Oncol.

[58] XW Naoko Matsuda, Krishnamurthy Savitri, Alvarez Ricardo H., Willey Jie S., Lim Bora, Parker Charla A., Babiera Gildy, Booser Daniel J., Murray James L., Arun Banu, Brewster Abenaa M., Reuben James M., Stauder Michael Charles, Woodward Wendy A., Lucci Anthony, DeSnyder Sarah Marie, Tripathy Debu, Valero Vicente, Ueno. Naoto T (2016). Phase II study of panitumumab, nab-paclitaxel, and carboplatin followed by FEC neoadjuvant chemotherapy for patients with primary HER2-negative inflammatory breast cancer. J Clin Oncol 34.

[59] Cristofanilli M, Gonzalez-Angulo AM, Buzdar AU, Kau SW, Frye DK, Hortobagyi GN (2004). Paclitaxel improves the prognosis in estrogen receptor negative inflammatory breast cancer: the M. D. Anderson Cancer Center experience. Clin Breast Cancer.

[60] Garcia S, Dales JP, Jacquemier J, Charafe-Jauffret E, Birnbaum D, Andrac-Meyer L, Lavaut MN, Allasia C, Carpentier-Meunier S, Bonnier P, Charpin-Taranger C (2007). c-Met overexpression in inflammatory breast carcinomas: automated quantification on tissue microarrays. Br J Cancer.

[61] Baselga J, Campone M, Piccart M, Burris HA, HS 3rd Rugo, Sahmoud T, Noguchi S, Gnant M, Pritchard KI, Lebrun F, Beck JT, Ito Y, Yardley D (2012). Everolimus in postmenopausal hormone-receptor-positive advanced breast cancer. N Engl J Med.

[62] Flatley E, Ang D, Warrick A, Beadling C, Corless CL, Troxell ML (2013). PIK3CA-AKT pathway mutations in micropapillary breast carcinoma. Hum Pathol.

[63] Stemke-Hale K, Gonzalez-Angulo AM, Lluch A, Neve RM, Kuo WL, Davies M, Carey M, Hu Z, Guan Y, Sahin A, Symmans WF, Pusztai L, Nolden LK (2008). An integrative genomic and proteomic analysis of PIK3CA, PTEN, and AKT mutations in breast cancer. Cancer Res.

[64] Shaw RJ, Cantley LC (2006). Ras, PI(3)K and mTOR signalling controls tumour cell growth. Nature.

[65] Liu P, Cheng H, Roberts TM, Zhao JJ (2009). Targeting the phosphoinositide 3-kinase pathway in cancer. Nat Rev Drug Discov.

[66] Jhaveri K, Teplinsky E, Silvera D, Valeta-Magara A, Arju R, Giashuddin S, Sarfraz Y, Alexander M, Darvishian F, Levine PH, Hashmi S, Zolfaghari L, Hoffman HJ (2016). Hyperactivated mTOR and JAK2/STAT3 Pathways: Molecular Drivers and Potential Therapeutic Targets of Inflammatory and Invasive Ductal Breast Cancers After Neoadjuvant Chemotherapy. Clin Breast Cancer.

[67] Austin L LK, Palazzo J, Avery T, Jaslow R, Hencin R, Petricoin EF, Cristofanilli M (2014). Identifying molecular targets and mechanisms of treatment resistance in inflammatory breast cancer (IBC) using reverse-phase protein microarrays (RPMA). SAn Antonio Breast Conference 2014 Poster Session: Tumor Heterogeneity/Molecular Subclassification (7: 30 AM-9: 00 AM) Abstract P2-04-02.

[68] Williams KP, Allensworth JL, Ingram SM, Smith GR, Aldrich AJ, Sexton JZ, Devi GR (2013). Quantitative high-throughput efficacy profiling of approved oncology drugs in inflammatory breast cancer models of acquired drug resistance and re-sensitization. Cancer Lett.

[69] Forozan F, Veldman R, Ammerman CA, Parsa NZ, Kallioniemi A, Kallioniemi OP, Ethier SP (1999). Molecular cytogenetic analysis of 11 new breast cancer cell lines. Br J Cancer.

[70] Andre F, O'Regan R, Ozguroglu M, Toi M, Xu B, Jerusalem G, Masuda N, Wilks S, Arena F, Isaacs C, Yap YS, Papai Z, Lang I (2014). Everolimus for women with trastuzumab-resistant, HER2-positive, advanced breast cancer (BOLERO-3): a randomised, double-blind, placebo-controlled phase 3 trial. Lancet Oncol.

[71] Relf M, LeJeune S, Scott PA, Fox S, Smith K, Leek R, Moghaddam A, Whitehouse R, Bicknell R, Harris AL (1997). Expression of the angiogenic factors vascular endothelial cell growth factor, acidic and basic fibroblast growth factor, tumor growth factor beta-1, platelet-derived endothelial cell growth factor, placenta growth factor, and pleiotrophin in human primary breast cancer and its relation to angiogenesis. Cancer Res.

[72] Dvorak HF (2002). Vascular permeability factor/vascular endothelial growth factor: a critical cytokine in tumor angiogenesis and a potential target for diagnosis and therapy. J Clin Oncol.

[73] Miller K, Wang M, Gralow J, Dickler M, Cobleigh M, Perez EA, Shenkier T, Cella D, Davidson NE (2007). Paclitaxel plus bevacizumab versus paclitaxel alone for metastatic breast cancer. N Engl J Med.

[74] Robert NJ, Dieras V, Glaspy J, Brufsky AM, Bondarenko I, Lipatov ON, Perez EA, Yardley DA, Chan SY, Zhou X, Phan SC, O'Shaughnessy J (2011). RIBBON-1: randomized, double-blind, placebo-controlled, phase III trial of chemotherapy with or without bevacizumab for first-line treatment of human epidermal growth factor receptor 2-negative, locally recurrent or metastatic breast cancer. J Clin Oncol.

[75] Colpaert CG, Vermeulen PB, Benoy I, Soubry A, van Roy F, van Beest P, Goovaerts G, Dirix LY, van Dam P, Fox SB, Harris AL, van Marck EA (2003). Inflammatory breast cancer shows angiogenesis with high endothelial proliferation rate and strong E-cadherin expression. Br J Cancer.

[76] Wedam SB, Low JA, Yang SX, Chow CK, Choyke P, Danforth D, Hewitt SM, Berman A, Steinberg SM, Liewehr DJ, Plehn J, Doshi A, Thomasson D (2006). Antiangiogenic and antitumor effects of bevacizumab in patients with inflammatory and locally advanced breast cancer. J Clin Oncol.

[77] Cristofanilli M, Buzdar AU, Sneige N, Smith T, Wasaff B, Ibrahim N, Booser D, Rivera E, Murray JL, Valero V, Ueno N, Singletary ES, Hunt K (2001). Paclitaxel in the multimodality treatment for inflammatory breast carcinoma. Cancer.

[78] Bertucci F, Fekih M, Autret A, Petit T, Dalenc F, Levy C, Romieu G, Bonneterre J, Ferrero JM, Kerbrat P, Soulie P, Mouret-Reynier MA, Bachelot T (2016). Bevacizumab plus neoadjuvant chemotherapy in patients with HER2-negative inflammatory breast cancer (BEVERLY-1): a multicentre, single-arm, phase 2 study. Lancet Oncol.

[79] Goncalves A, Pierga JY, Ferrero JM, Mouret-Reynier MA, Bachelot T, Delva R, Fabbro M, Lerebours F, Lotz JP, Linassier C, Dohollou N, Eymard JC, Leduc B (2015). UNICANCER-PEGASE 07 study: a randomized phase III trial evaluating postoperative docetaxel-5FU regimen after neoadjuvant dose-intense chemotherapy for treatment of inflammatory breast cancer. Ann Oncol.

[80] Pierga JY, Petit T, Delozier T, Ferrero JM, Campone M, Gligorov J, Lerebours F, Roche H, Bachelot T, Charafe-Jauffret E, Pavlyuk M, Kraemer S, Bidard FC (2012). Neoadjuvant bevacizumab, trastuzumab, and chemotherapy for primary inflammatory HER2-positive breast cancer (BEVERLY-2): an open-label, single-arm phase 2 study. Lancet Oncol.

[81] Dawood S, Gong Y, Broglio K, Buchholz TA, Woodward W, Lucci A, Valero V, Gonzalez-Angulo AM, Hortobagyi GN, Cristofanilli M (2010). Trastuzumab in Primary Inflammatory Breast Cancer (IBC): High Pathological Response Rates and Improved Outcome. Breast J.

[82] Kumar R, Knick VB, Rudolph SK, Johnson JH, Crosby RM, Crouthamel MC, Hopper TM, Miller CG, Harrington LE, Onori JA, Mullin RJ, Gilmer TM, Truesdale AT (2007). Pharmacokinetic-pharmacodynamic correlation from mouse to human with pazopanib, a multikinase angiogenesis inhibitor with potent antitumor and antiangiogenic activity. Mol Cancer Ther.

[83] Hurwitz HI, Dowlati A, Saini S, Savage S, Suttle AB, Gibson DM, Hodge JP, Merkle EM, Pandite L (2009). Phase I trial of pazopanib in patients with advanced cancer. Clin Cancer Res.

[84] Cristofanilli M, Johnston SR, Manikhas A, Gomez HL, Gladkov O, Shao Z, Safina S, Blackwell KL, Alvarez RH, Rubin SD, Ranganathan S, Redhu S, Trudeau ME (2013). A randomized phase II study of lapatinib + pazopanib versus lapatinib in patients with HER2+ inflammatory breast cancer. Breast Cancer Res Treat.

[85] Quintas-Cardama A, Verstovsek S (2013). Molecular pathways: Jak/STAT pathway: mutations, inhibitors, and resistance. Clin Cancer Res.

[86] Heinrich PC, Behrmann I, Muller-Newen G, Schaper F, Graeve L (1998). Interleukin-6-type cytokine signalling through the gp130/Jak/STAT pathway. Biochem J.

[87] Sansone P, Bromberg J (2012). Targeting the interleukin-6/Jak/stat pathway in human malignancies. J Clin Oncol.

[88] Berishaj M, Gao SP, Ahmed S, Leslie K, Al-Ahmadie H, Gerald WL, Bornmann W, Bromberg JF (2007). Stat3 is tyrosine-phosphorylated through the interleukin-6/glycoprotein 130/Janus kinase pathway in breast cancer. Breast Cancer Res.

[89] Diaz N, Minton S, Cox C, Bowman T, Gritsko T, Garcia R, Eweis I, Wloch M, Livingston S, Seijo E, Cantor A, Lee JH, Beam CA (2006). Activation of stat3 in primary tumors from high-risk breast cancer patients is associated with elevated levels of activated SRC and survivin expression. Clin Cancer Res.

[90] Bouchard C, Dittrich O, Kiermaier A, Dohmann K, Menkel A, Eilers M, Luscher B (2001). Regulation of cyclin D2 gene expression by the Myc/Max/Mad network: Myc-dependent TRRAP recruitment and histone acetylation at the cyclin D2 promoter. Genes Dev.

[91] Hermeking H, Rago C, Schuhmacher M, Li Q, Barrett JF, Obaya AJ, O'Connell BC, Mateyak MK, Tam W, Kohlhuber F, Dang CV, Sedivy JM, Eick D (2000). Identification of CDK4 as a target of c-MYC. Proc Natl Acad Sci U S A.

[92] Obaya AJ, Kotenko I, Cole MD, Sedivy JM (2002). The proto-oncogene c-myc acts through the cyclin-dependent kinase (Cdk) inhibitor p27(Kip1) to facilitate the activation of Cdk4/6 and early G(1) phase progression. J Biol Chem.

[93] O'Donnell KA, Wentzel EA, Zeller KI, Dang CV, Mendell JT (2005). c-Myc-regulated microRNAs modulate E2F1 expression. Nature.

[94] Ross JS, Ali SM, Wang K, Khaira D, Palma NA, Chmielecki J, Palmer GA, Morosini D, Elvin JA, Fernandez SV, Miller VA, Stephens PJ, Cristofanilli M (2015). Comprehensive genomic profiling of inflammatory breast cancer cases reveals a high frequency of clinically relevant genomic alterations. Breast Cancer Res Treat.

[95] Finn RS, Dering J, Conklin D, Kalous O, Cohen DJ, Desai AJ, Ginther C, Atefi M, Chen I, Fowst C, Los G, Slamon DJ (2009). PD 0332991, a selective cyclin D kinase 4/6 inhibitor, preferentially inhibits proliferation of luminal estrogen receptor-positive human breast cancer cell lines in vitro. Breast Cancer Res.

[96] van Golen KL, Davies S, Wu ZF, Wang Y, Bucana CD, Root H, Chandrasekharappa S, Strawderman M, Ethier SP, Merajver SD (1999). A novel putative low-affinity insulin-like growth factor-binding protein, LIBC (lost in inflammatory breast cancer), and RhoC GTPase correlate with the inflammatory breast cancer phenotype. Clin Cancer Res.

[97] van Golen KL, Wu ZF, Qiao XT, Bao LW, Merajver SD (2000). RhoC GTPase, a novel transforming oncogene for human mammary epithelial cells that partially recapitulates the inflammatory breast cancer phenotype. Cancer Res.

[98] Wu M, Wu ZF, Rosenthal DT, Rhee EM, Merajver SD (2010). Characterization of the roles of RHOC and RHOA GTPases in invasion, motility, and matrix adhesion in inflammatory and aggressive breast cancers. Cancer.

[99] van Golen KL, Bao L, DiVito MM, Wu Z, Prendergast GC, Merajver SD (2002). Reversion of RhoC GTPase-induced inflammatory breast cancer phenotype by treatment with a farnesyl transferase inhibitor. Mol Cancer Ther.

[100] Andreopoulou E, Vigoda IS, Valero V, Hershman DL, Raptis G, Vahdat LT, Han HS, Wright JJ, Pellegrino CM, Cristofanilli M, Alvarez RH, Fehn K, Fineberg S (2013). Phase I-II study of the farnesyl transferase inhibitor tipifarnib plus sequential weekly paclitaxel and doxorubicin-cyclophosphamide in HER2/neu-negative inflammatory carcinoma and non-inflammatory estrogen receptor-positive breast carcinoma. Breast Cancer Res Treat.

[101] Sparano JA, Moulder S, Kazi A, Coppola D, Negassa A, Vahdat L, Li T, Pellegrino C, Fineberg S, Munster P, Malafa M, Lee D, Hoschander S (2009). Phase II trial of tipifarnib plus neoadjuvant doxorubicin-cyclophosphamide in patients with clinical stage IIB-IIIC breast cancer. Clin Cancer Res.

[102] Lehman HL, Van Laere SJ, van Golen CM, Vermeulen PB, Dirix LY, van Golen KL (2012). Regulation of inflammatory breast cancer cell invasion through Akt1/PKBalpha phosphorylation of RhoC GTPase. Mol Cancer Res.

[103] Soda M, Choi YL, Enomoto M, Takada S, Yamashita Y, Ishikawa S, Fujiwara S, Watanabe H, Kurashina K, Hatanaka H, Bando M, Ohno S, Ishikawa Y (2007). Identification of the transforming EML4-ALK fusion gene in non-small-cell lung cancer. Nature.

[104] Solomon BJ, Mok T, Kim DW, Wu YL, Nakagawa K, Mekhail T, Felip E, Cappuzzo F, Paolini J, Usari T, Iyer S, Reisman A, Wilner KD (2014). First-line crizotinib versus chemotherapy in ALK-positive lung cancer. N Engl J Med.

[105] Shaw AT, Gandhi L, Gadgeel S, Riely GJ, Cetnar J, West H, Camidge DR, Socinski MA, Chiappori A, Mekhail T, Chao BH, Borghaei H, Gold KA (2016). Alectinib in ALK-positive, crizotinib-resistant, non-small-cell lung cancer: a single-group, multicentre, phase 2 trial. Lancet Oncol.

[106] Fukuyoshi Y, Inoue H, Kita Y, Utsunomiya T, Ishida T, Mori M (2008). EML4-ALK fusion transcript is not found in gastrointestinal and breast cancers. Br J Cancer.

[107] Robertson FM, EF Petricoin Iii, Van Laere SJ, Bertucci F, Chu K, Fernandez SV, Mu Z, Alpaugh K, Pei J, Circo R, Wulfkuhle J, Ye Z, Boley KM (2013). Presence of anaplastic lymphoma kinase in inflammatory breast cancer. Springerplus.

[108] Colpaert C MM, Vermeulen P, Dirix L, Van Laere S Inflammatory Breast Cancer International Consortium. [P3-06-39] Anaplastic lymphoma kinase (ALK) protein overexpression is not a feature of inflammatory breast cancer. San Antonio Breast Conference.

[109] Vire E, Brenner C, Deplus R, Blanchon L, Fraga M, Didelot C, Morey L, Van Eynde A, Bernard D, Vanderwinden JM, Bollen M, Esteller M, Di Croce L (2006). The Polycomb group protein EZH2 directly controls DNA methylation. Nature.

[110] Gong Y, Huo L, Liu P, Sneige N, Sun X, Ueno NT, Lucci A, Buchholz TA, Valero V, Cristofanilli M (2011). Polycomb group protein EZH2 is frequently expressed in inflammatory breast cancer and is predictive of worse clinical outcome. Cancer.

[111] Mu Z, Li H, Fernandez SV, Alpaugh KR, Zhang R, Cristofanilli M (2013). EZH2 knockdown suppresses the growth and invasion of human inflammatory breast cancer cells. J Exp Clin Cancer Res.

[112] Robertson FM, Chu K, Boley KM, Ye Z, Liu H, Wright MC, Moraes R, Zhang X, Green TL, Barsky SH, Heise C, Cristofanilli M (2013). The class I HDAC inhibitor Romidepsin targets inflammatory breast cancer tumor emboli and synergizes with paclitaxel to inhibit metastasis. J Exp Ther Oncol.

[113] Avery TP JR, Basu-Mallick A, Zibelli A, Fellin F, Cristofanilli M, Philadelphia PA, Thomas Jefferson University [P4-13-18] A phase I study of romidepsin in combination with nab-paclitaxel in patients with metastatic HER-2 negative inflammatory breast cancer (IBC). San Antonio Breast Conference.

[114] Torres-Adorno AM LJ, Kogawa T, Bartholomeusz C, Pitner MK, Ordentlich P, Lim B, Tripathy D, Ueno NT [P5-04-02] The histone deacetylase inhibitor entinostat enhances the efficacy of the MEK inhibitor pimasertib against aggressive types of breast cancer through Noxa-mediated myeloid cell leukemia 1 degradation. San Antonio Breast Conference 2015 Abstract [P2-04-02].

[115] Chatterjee N, Wang WL, Conklin T, Chittur S, Tenniswood M (2013). Histone deacetylase inhibitors modulate miRNA and mRNA expression, block metaphase, and induce apoptosis in inflammatory breast cancer cells. Cancer Biol Ther.

[116] Brahmer J, Reckamp KL, Baas P, Crino L, Eberhardt WE, Poddubskaya E, Antonia S, Pluzanski A, Vokes EE, Holgado E, Waterhouse D, Ready N, Gainor J (2015). Nivolumab versus Docetaxel in Advanced Squamous-Cell Non-Small-Cell Lung Cancer. N Engl J Med.

[117] Borghaei H, Paz-Ares L, Horn L, Spigel DR, Steins M, Ready NE, Chow LQ, Vokes EE, Felip E, Holgado E, Barlesi F, Kohlhaufl M, Arrieta O (2015). Nivolumab versus Docetaxel in Advanced Nonsquamous Non-Small-Cell Lung Cancer. N Engl J Med.

[118] Motzer RJ, Escudier B, McDermott DF, George S, Hammers HJ, Srinivas S, Tykodi SS, Sosman JA, Procopio G, Plimack ER, Castellano D, Choueiri TK, Gurney H (2015). Nivolumab versus Everolimus in Advanced Renal-Cell Carcinoma. N Engl J Med.

[119] Wolchok JD, Kluger H, Callahan MK, Postow MA, Rizvi NA, Lesokhin AM, Segal NH, Ariyan CE, Gordon RA, Reed K, Burke MM, Caldwell A, Kronenberg SA (2013). Nivolumab plus ipilimumab in advanced melanoma. N Engl J Med.

[120] Wang C, Thudium KB, Han M, Wang XT, Huang H, Feingersh D, Garcia C, Wu Y, Kuhne M, Srinivasan M, Singh S, Wong S, Garner N (2014). In vitro characterization of the anti-PD-1 antibody nivolumab, BMS-936558, and in vivo toxicology in non-human primates. Cancer Immunol Res.

[121] Champiat S, Ferte C, Lebel-Binay S, Eggermont A, Soria JC (2014). Exomics and immunogenics: Bridging mutational load and immune checkpoints efficacy. Oncoimmunology.

[122] Rosenberg SA (2014). Decade in review-cancer immunotherapy: entering the mainstream of cancer treatment. Nat Rev Clin Oncol.

[123] JN Le DT Uram, Wang H, Bartlett BR, Kemberling H, Eyring AD, Skora AD, Luber BS, Azad NS, Laheru D, Biedrzycki B, Donehower RC, Zaheer A (2015). PD-1 Blockade in Tumors with Mismatch-Repair Deficiency. N Engl J Med.

[124] Nanda R, Chow LQ, Dees EC, Berger R, Gupta S, Geva R, Pusztai L, Pathiraja K, Aktan G, Cheng JD, Karantza V, Buisseret L (2016). Pembrolizumab in Patients With Advanced Triple-Negative Breast Cancer: Phase Ib KEYNOTE-012 Study. J Clin Oncol.

[125] Dirix LY TI, Nikolinakos P, Jerusalem G, Arkenau H-T, Hamilton EP, von Heydebreck A, Grote H-J, Chin K, Lippman ME, Sint Augustinus (2015). Avelumab (MSB0010718C), an anti-PD-L1 antibody, in patients with locally advanced or metastatic breast cancer: A phase Ib JAVELIN solid tumor trial. San Antonio Breast Conference [S1-04] 2015.

[126] Bertucci F, Finetti P, Colpaert C, Mamessier E, Parizel M, Dirix L, Viens P, Birnbaum D, van Laere S (2015). PDL1 expression in inflammatory breast cancer is frequent and predicts for the pathological response to chemotherapy. Oncotarget.

[127] Rea D, Francis A, Hanby AM, Speirs V, Rakha E, Shaaban A, Chan S, Vinnicombe S, Ellis IO, Martin SG, Jones LJ, Berditchevski F (2015). Inflammatory breast cancer: time to standardise diagnosis assessment and management, and for the joining of forces to facilitate effective research. Br J Cancer.

[128] Bertucci F, Ueno NT, Finetti P, Vermeulen P, Lucci A, Robertson FM, Marsan M, Iwamoto T, Krishnamurthy S, Masuda H, Van Dam P, Woodward WA, Cristofanilli M (2014). Gene expression profiles of inflammatory breast cancer: correlation with response to neoadjuvant chemotherapy and metastasis-free survival. Ann Oncol.

